# SNAIL is a key regulator of alveolar rhabdomyosarcoma tumor growth and differentiation through repression of MYF5 and MYOD function

**DOI:** 10.1038/s41419-018-0693-8

**Published:** 2018-05-29

**Authors:** Klaudia Skrzypek, Anna Kusienicka, Elzbieta Trzyna, Barbara Szewczyk, Aleksandra Ulman, Pawel Konieczny, Tomasz Adamus, Bogna Badyra, Marcin Kortylewski, Marcin Majka

**Affiliations:** 1 Department of Transplantation, Institute of Pediatrics, Jagiellonian University Medical College, Wielicka 265, Krakow, 30-663 Poland; 2 Department of Onco-Immunology, Beckman Research Institute at City of Hope National Medical Center, Duarte, CA 91010 USA

## Abstract

Rhabdomyosarcoma (RMS) is a mesenchymal tumor of soft tissue in children that originates from a myogenic differentiation defect. Expression of SNAIL transcription factor is elevated in the alveolar subtype of RMS (ARMS), characterized by a low myogenic differentiation status and high aggressiveness. In RMS patients SNAIL level increases with higher stage. Moreover, SNAIL level negatively correlates with MYF5 expression. The differentiation of human ARMS cells diminishes SNAIL level. SNAIL silencing in ARMS cells inhibits proliferation and induces differentiation *in vitro*, and thereby completely abolishes the growth of human ARMS xenotransplants *in vivo*. SNAIL silencing induces myogenic differentiation by upregulation of myogenic factors and muscle-specific microRNAs, such as miR-206. SNAIL binds to the MYF5 promoter suppressing its expression. SNAIL displaces MYOD from E-box sequences (CANNTG) that are associated with genes expressed during differentiation and G/C rich in their central dinucleotides. SNAIL silencing allows the re-expression of MYF5 and canonical MYOD binding, promoting ARMS cell myogenic differentiation. In differentiating ARMS cells SNAIL forms repressive complex with histone deacetylates 1 and 2 (HDAC1/2) and regulates their expression. Accordingly, in human myoblasts SNAIL silencing induces differentiation by upregulation of myogenic factors. Our data clearly point to SNAIL as a key regulator of myogenic differentiation and a new promising target for future ARMS therapies.

## Introduction

Rhabdomyosarcoma (RMS) is the most frequently occurring soft tissue sarcoma among children and adolescents, however, rare instances of the disease have been noted in adults. Based on histological analysis of the tumor, two major RMS subtypes may be distinguished: embryonal (ERMS) and alveolar (ARMS). ERMS is usually associated with better survival and typically occurs in the head and neck and urogenital tract. ARMS occurs in the extremities and trunk and generally has a significantly worse prognosis^1–3^. High aggressiveness of ARMS subtype is associated with presence of PAX3-FOXO1 or PAX7-FOXO1 fusion genes and increased levels of MET receptor, a member of tyrosine kinase receptors family (RTK), which is associated with metastatic potential of RMS cells^2,4^. RMS development is likely connected to a differentiation defect of stem cells or early progenitors, such as mesenchymal stem cells or satellite cells/myoblasts^3,5,6^.

Myogenic differentiation is regulated by different early and late myogenic factors. These basic helix-loop-helix (bHLH) transcription activators contain a conserved DNA binding domain, that recognizes the enhancer box (E-box) motif[7]. Myogenic factor 5 (MYF5) and myogenic differentiation 1 (MYOD/MYOD1) are responsible for the early stages of differentiation, whereas myogenin (MYOG) and myogenic factor 6 (MRF4) are responsible for terminal differentiation^7^. Those myogenic factors regulate genes and muscle-specific microRNAs. An interesting example is miR-206, which is induced by MYOD to enhance differentiation and facilitate cell cycle exit^8^. Aberrations within the regulatory myogenic pathway described above may be one of the crucial causes of the rhabdomyosarcoma development^3,4^.

In our previous work, we observed that aggressive ARMS tumors display higher expression of the SNAIL gene and that SNAIL expression positively correlates with PAX3/7-FOXO1^9^. SNAIL (snail family zinc finger 1; SNAI1) is a member of the group of zinc finger transcription factors. The SNAIL family consists of 3 members: SNAIL (SNAI1), SLUG (SNAI2) and SMUG (SNAI3). SNAIL plays an eminent role in the epithelial to mesenchymal transition (EMT), the main mechanism responsible for both embryogenesis and the invasiveness and metastasis of neoplasms^10–12^. By binding to target E-box sequences (CANNTG), SNAIL acts as a transcriptional repressor, but it may also work as a gene activator^13^. SNAIL, causes epigenetic changes in chromatin structure, mainly by recruiting histone deacetylases (HDACs)^14^.

The role of SNAIL in non-epithelial tumors is poorly understood. Recent data have provided evidence that SNAIL plays an important role in cells of mesenchymal origin. Murine fibroblasts expressing SNAIL in epithelial tumor microenvironment support metastasis, given their mechanical properties^15^. Moreover, when SNAIL expression is reactivated in mesenchymal cells, they may become invasive, whereas SNAIL-deficient fibroblasts do not display invasive capabilities^16^. Activated mesenchymal cells display SNAIL expression, and SNAIL seems to play an important role in the communication between the stroma and the tumor and it can facilitate the conversion of cancer cells to stromal cells^17^. In sarcomas and fibrosarcomas, SNAIL levels are elevated^17^. However, the precise mechanism of its action remains unknown.

The mechanistic role of SNAIL in the biology of ARMS has not been investigated, although, the important role of SNAIL in murine myogenesis has been described previously^18^. SNAIL zinc-finger binding domains recognize the same E-box sequence as bHLH myogenic transcription factors. The repressive complex of SNAIL with HDACs binds and excludes MYOD from G/C-rich E-box motifs in murine satellite cells, thereby inhibiting myogenic differentiation. In RMS SNAIL expression is higher in ARMS tumor^9^ that is associated with worse prognosis and SNAIL is a mediator of NOTCH pathway in ERMS subtype^19^.

Here, we demonstrate for the first time that SNAIL blocks human ARMS differentiation and promotes tumor development. Importantly, our study describes the molecular mechanism of SNAIL, which deregulates growth and myogenic differentiation of ARMS cells through interference with the early myogenic transcription factors, MYF5 and MYOD.

## Results

### SNAIL expression is associated with RMS myogenic differentiation

**Fig. 1:**
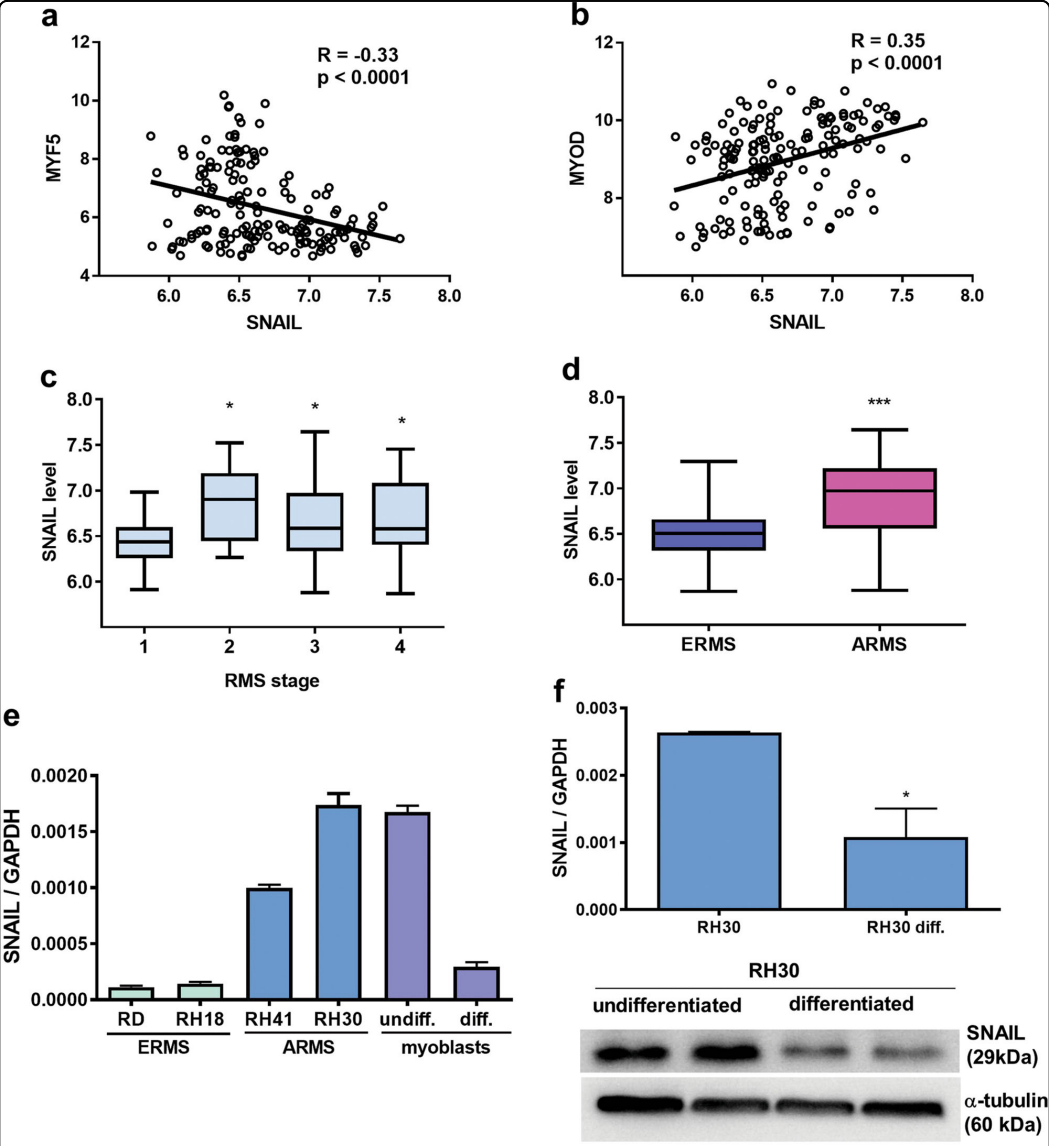
SNAIL expression is associated with RMS subtype and myogenic differentiation. **a** Expression of early and late myogenic factors in 158 RMS samples was estimated previously by microarray and deposited in GEO database with accession number: GSE92689 (ref. ^20^). SNAIL level negatively correlates with MYF5 level in RMS (Pearson correlation). **b** SNAIL level positively correlates with MYOD level in RMS (Pearson correlation). R—Pearson correlation coefficient; p—significance value (**c**) Increased SNAIL levels in RMS stage 2,3 and 4 compared to 1 were demonstrated in a group of 158 RMS samples (microarray data, GEO database GSE92689). The data are presented as Whisker plots min to max. **d** Increased SNAIL level in ARMS compared to ERMS was demonstrated in a group of 158 RMS samples (microarray data, GEO database GSE92689). The data are presented as Whisker plots min to max. **e** SNAIL expression was quantified by qPCR using the ΔCt quantification method and GAPDH as a housekeeping gene control in ARMS and ERMS cell lines and human primary myoblasts. The SNAIL level is increased in RH30 and RH41 ARMS cell lines compared to RD and RH18 ERMS cell lines, and the level is comparable with the SNAIL level in myoblasts at the early passage (undiff.), whereas in the differentiated myotubes (diff.) SNAIL level is more comparable with ERMS; *n* = 3. **f** Differentiation of RH30 cells by culture in medium supplemented with 2% HS for 7 days downregulates SNAIL expression at the mRNA level (qPCR) and protein level (Western blotting); *n* = 4. The data represent the mean ± SEM. *p < 0.05, **p < 0.01, ***p < 0.001

Because RMS can originate from the impaired differentiation of myogenic progenitors, we analyzed the expression of myogenic regulatory factors and SNAIL in patient samples obtained from two major RMS subtypes, ERMS and ARMS. Bioinformatical analysis of microarray data deposited in GEO database (ref. ^20^) was performed according to literature^21^ and revealed that in 158 RMS patients samples SNAIL level negatively correlated with MYF5 level (Fig. 1a) and positively correlated with MYOD (Fig. 1b). In a group of 158 samples SNAIL level turned out to be significantly increased in RMS samples from patients displaying the stages of the disease 2, 3, and 4 compared to stage 1, what suggested an important role of SNAIL in RMS progression (Fig. 1c). Moreover, SNAIL level was elevated in ARMS compared to ERMS (Fig. 1d), what is in accordance with the staging results, because ARMS is not included in stage 1 due to more unfavorable histology. Those data indicated that SNAIL may play an important role in the development of less differentiated ARMS tumors that are associated with worse prognosis.

SNAIL levels were also significantly increased in RH41 and RH30 ARMS cell lines compared with RD and RH18 ERMS cell lines, and the levels were comparable with the SNAIL level in human myoblasts at early passages, whereas differentiated myotubes displayed the diminished SNAIL levels that were comparable with ERMS cells (Fig. 1e). Forced myogenic differentiation of RH30 ARMS cells for 7 days significantly diminished SNAIL expression at the mRNA and protein levels (Fig. 1f). These results suggested the direct association of SNAIL expression with myogenic differentiation.

### SNAIL silencing changes the ARMS phenotype and completely inhibits tumor growth in mice

**Fig. 2:**
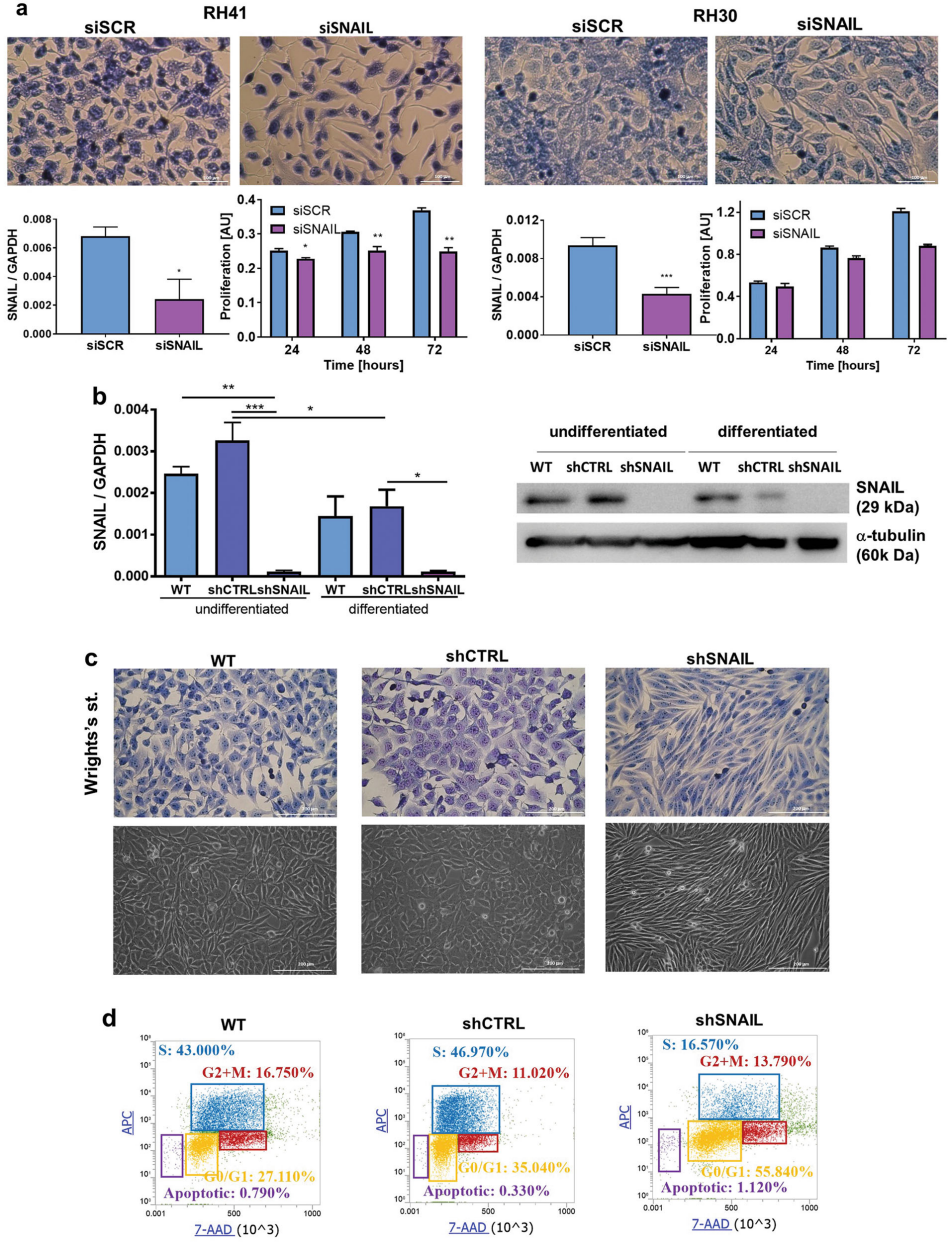
SNAIL silencing in ARMS cells induces a spindle-shaped phenotype and inhibits proliferation. **a** SNAIL silencing by siRNA induces the acquisition of spindle-shaped phenotype of RH41 and RH30 cells and diminishes their proliferation. RH30 ARMS cells were transfected with siRNA against SNAIL (siSNAIL) and a scrambled siRNA sequence (siRNA). 24 h after transfection medium was changed for a differentiating medium containing 2% HS for the next 2 days. SNAIL silencing with siRNA for 3 days was validated by qPCR; *n* = 4. The morphology of the cells was visualized with Wright’s staining (error bar represent 100 μm). Proliferation was evaluated by MTS assay; *n* = 3. **b** To stably silence the SNAIL level, RH30 cells were transduced with shRNA lentiviral vectors targeting SNAIL (shSNAIL) and control vectors (shCTRL), and these cells were selected with puromycin. SNAIL silencing was validated by qPCR (*n* = 3) and Western blotting (total cell extracts). **c** Stable SNAIL silencing in RH30 cells leads to the acquisition of a spindle- shaped phenotype and reorganization of the cytoskeleton. The morphology of RH30 cells differentiated for 4 days was visualized with Wright’s staining and for 6 days with phase contrast microscopy. **d** SNAIL silencing and the differentiation of RH30 cells led to cell cycle arrest at the G0-G1 phase and diminished percentage of the cells in S phase. RH30 cells were differentiated for 7 days in medium containing 2% HS. Subsequently, the cell cycle and BrdU incorporation in S phase were analyzed by flow cytometry—the cells were stained with anti-BrdU antibody conjugated with APC and with 7AAD. The data in the graphs represent the mean ± SEM or are representative images of at least 3 independent experiments. *p < 0.05, **p < 0.01, ***p < 0.001

Since ARMS tumors and cell lines display increased SNAIL levels, we silenced SNAIL with siRNA in RH41 and RH30 ARMS cells and treated the cells with differentiating medium for the next 2 days. Transient SNAIL silencing diminished the cell proliferation and induced the acquisition of a more elongated (spindle-shaped) phenotype (Fig. 2a). Moreover, two different siRNA sequences against SNAIL resulted in acquisition of the elongated phenotype (spindle shape) in RH30 and RH41 cells (Supplementary Figure 1). Next, we stably silenced SNAIL by transduction of RH30 cells with mixture of three shRNA vectors (shSNAIL cells). Control cells were modified with scrambled shRNA (shCTRL). In the stable cell line SNAIL mRNA and protein downregulation was verified (Fig. 2b). Stable SNAIL silencing and cell culture in differentiating medium for 6 days resulted in morphological changes and the acquisition of a strongly elongated and polarized phenotype, resembling myotubes (Fig. 2c). The differentiation of RH30 shSNAIL cells for 7 days diminished proliferation and resulted in growth arrest at the G0/G1 phase and the diminished percentage of cells in S phase (Fig. 2d).

**Fig. 3:**
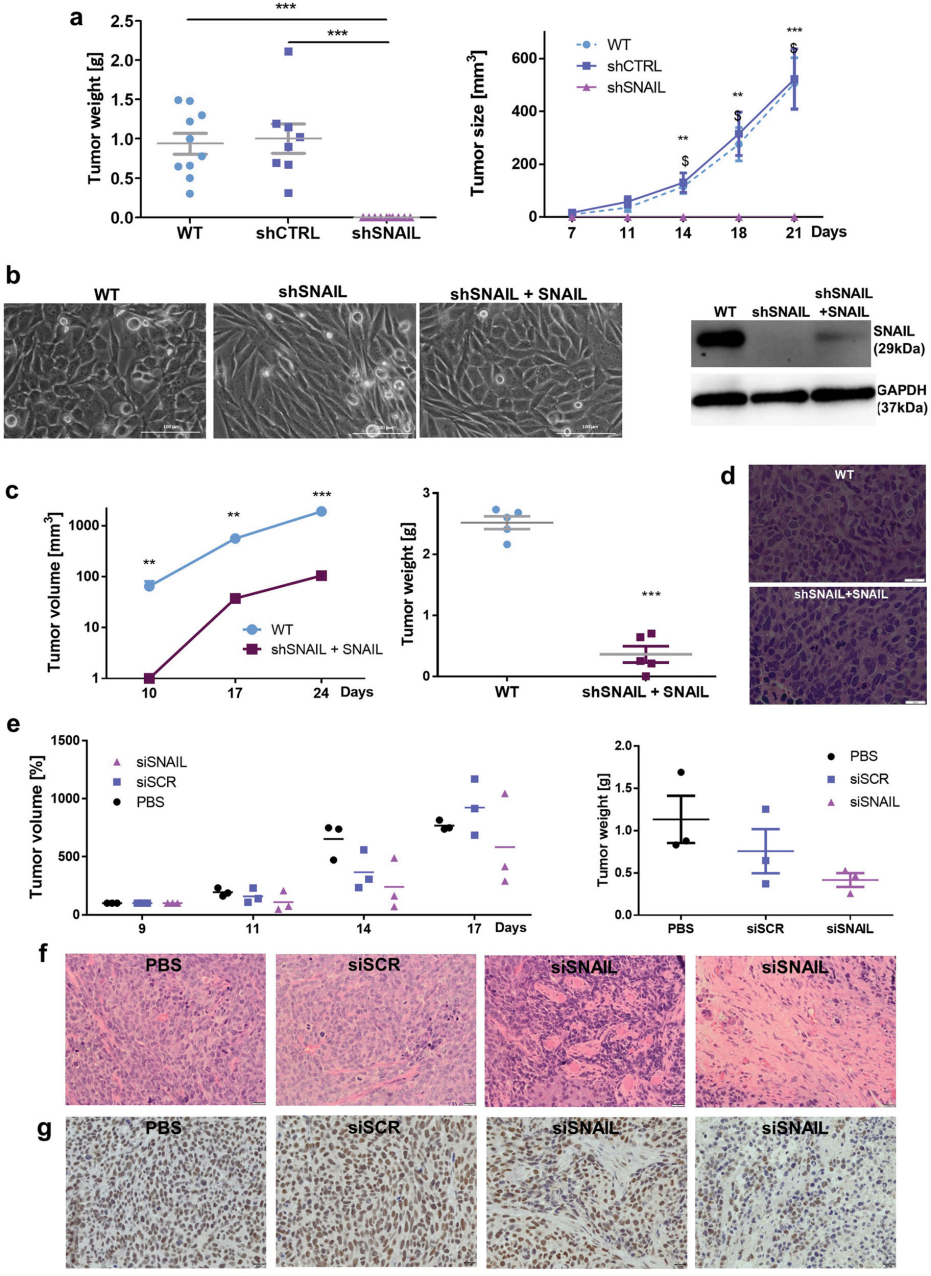
SNAIL silencing completely blocks the growth of RH30 ARMS xenotransplants in NOD-SCID mice. **a** In total, 5 × 10^6^ RH30 cells were subcutaneously implanted into immunodeficient NOD-SCID mice, and tumor growth was investigated for 3–4 weeks and measured with a caliper. RH30 shSNAIL cells do not form any tumors in NOD-SCID mice, whereas WT and shCTRL cells form tumors of approximately 1 g in weight and 600 cm^3^ in size within 21 days; *n* = 9–10. **b** Partial restoration of the SNAIL level by transduction with lentiviral vectors encoding SNAIL partially restores the phenotype of RH30 cells. The morphology of the cells was visualized by phase contrast microscopy, and SNAIL level was evaluated by Western blot. **c** Partial restoration of the SNAIL level leads to the appearance of tumors that are significantly smaller; *n* = 5. **d** RH30 tumors with partial restoration of the SNAIL level displayed similar morphology to tumors formed by WT cells (error bar represents 20 μm) **e** Tumor therapy with SNAIL siRNA slightly tends to diminish tumor growth. 5 × 10^6^ RH30 cells were subcutaneously implanted into immunodeficient NOD-SCID mice. Next, three injections of PBS, scrambled siRNA (siSCR) or SNAIL siRNA (siSNAIL) at dose 3 μg/kg to growing tumors were performed 9, 11, and 14 days after implantation of the cells. Tumor volume was evaluated with caliper until day 17 and it was calculated as percentage of volume from the start of the therapy. Weight of tumor was analyzed 17 days after the implantation. Each experimental group comprised of 3 mice. **f** Tumor therapy with SNAIL siRNA resulted in appearance of differentiated tumor morphology (visualized with hematoxylin-eosin staining). **g** SNAIL expression level in tumors treated with SNAIL siRNA was diminished in the regions displaying differentiating morphology (visualized with immunohistochemical staining of FFPE sections; error bar represents 20 μm). The data in the graphs represent the mean ± SEM or are representative images. *p < 0.05, **p < 0.01, ***p < 0.001

To investigate the effect of SNAIL silencing *in vivo*, we subcutaneously implanted RH30 cells into immunodeficient NOD-SCID mice, and we evaluated tumor growth for 21 days. SNAIL silencing completely abolished the development of any tumors, whereas wild type (WT) and control (shCTRL) tumors grew in mice, reaching up to 600 mm^3^ in size and 1 to 1.5 g in weight (Fig. 3a). Those results indicated for the first time that SNAIL is a crucial factor that regulates RMS growth.

To confirm that the observed effects are SNAIL dependent, we transduced RH30 shSNAIL cells with lentiviral vectors encoding GFP-P2A-SNAIL, resulting in the partial restoration of SNAIL protein expression and incomplete reversal of the phenotype (Fig. 3b). Subsequently, we implanted these cells into mice. Tumor development was noted within 28 days after implantation. However, these tumors were significantly smaller than the control tumors probably because the SNAIL level was not totally restored in these cells (Fig. 3c). Both tumor types displayed similar morphology (Fig. 3d), suggesting that only cells with a restored SNAIL level could form tumors in mice. To model clinical effect of SNAL silencing, we performed three injections of 3 μg/kg siRNA against SNAIL into growing RH30 tumors. The antitumor effect of SNAIL siRNA treatment tended to be visible at the end of therapy (Fig. 3e). Interestingly, the therapy resulted in appearance of differentiated morphology of tumors treated with SNAIL siRNA (Fig. 3f) and those regions displayed diminished SNAIL expression (Fig. 3g).

### SNAIL silencing in ARMS directly upregulates MYF5 and excludes MYOD from its DNA binding sites

**Fig. 4:**
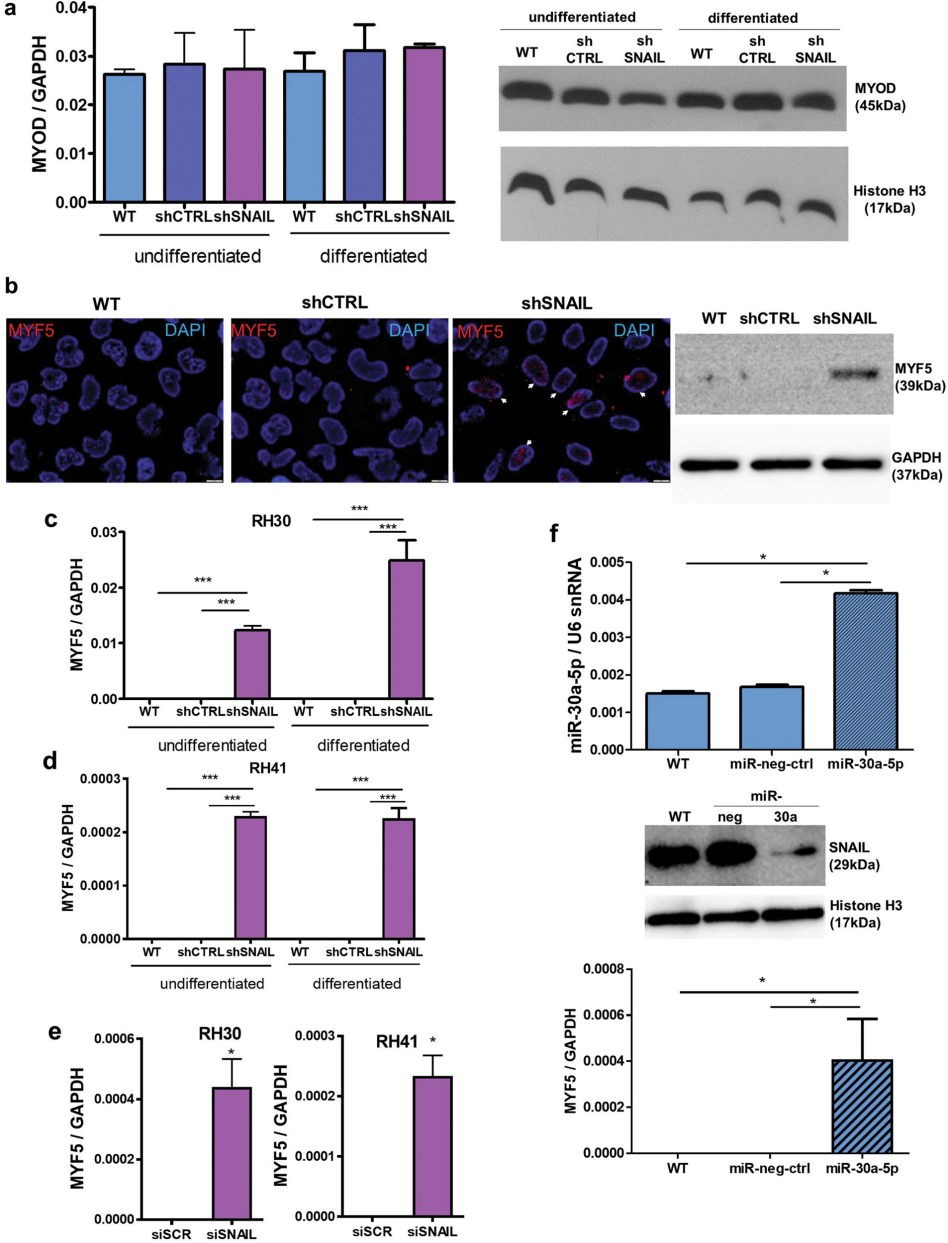
SNAIL silencing induces MYF5 expression in ARMS cells. **a** SNAIL silencing does not significantly affect MYOD expression at the mRNA (qPCR, *n* = 3) or protein level (Western blot) in undifferentiated RH30 cells and RH30 cells differentiated for 7 days in medium with 2% HS. **b** MYF5 is expressed in the nuclei of RH30 shSNAIL cells. The MYF5 protein level was visualized by Western blot and by immunofluorescent staining (red color), and the nuclei were visualized with DAPI (blue). The results are presented as merged images. The white scale bar represents 10 μm. **c** SNAIL silencing induces the expression of MYF5 mRNA in both undifferentiated RH30 cells and RH30 cells differentiated for 7 days in medium with 2% HS (qPCR, *n* = 3) **d** SNAIL silencing slightly induces the expression of MYF5 mRNA in both undifferentiated RH41 cells and RH41 cells differentiated for 7 days in medium with 2% HS (qPCR, *n* = 3). **e** Transient SNAIL silencing with siRNA in RH30 and RH41 ARMS cell lines slightly induces MYF5 expression. The cells were transfected with siRNA against SNAIL (siSNAIL) and a scrambled siRNA sequence (siRNA). Twenty-four hours after transfection, the cells were treated with differentiating medium containing 2% HS for the following 48 h. MYF5 levels were validated by qPCR; *n* = 3. **f** Transfection with pre-miR-30a-5p in RH30 cells induces MYF5 expression by downregulation of SNAIL protein. RH30 cells were transfected with the miRNA precursor pre-miR-30a-5p and pre-miR negative control (miR-neg-ctrl). Twenty-four hours after transfection, the cells were treated with differentiating medium containing 2% HS for the following 48 h. miR-30a-5p expression relative to U6 snRNA and MYF5, MYOD expression relative to GAPDH were validated by qPCR; *n* = 4. SNAIL protein level was validated by Western blot. The data represent the mean ± SEM. *p < 0.05, **p < 0.01, ***p < 0.001

Since expression of SNAIL negatively correlated with expression of myogenic factors, we hypothesized that complete inhibition of tumor growth *in vivo* may be a result of the myogenic differentiation of the cells. To evaluate this hypothesis, we investigated the effect of SNAIL on different early and late myogenic factors. SNAIL did not exert the significant effect on MYOD expression (Fig. 4a). Interestingly, the nuclei of RH30 shSNAIL cells displayed strong MYF5 expression, whereas control cells did not express MYF5 what was confirmed by Western blot analysis (Fig. 4b). Similarly, MYF5 mRNA was not expressed in RH30 cells, but SNAIL silencing strongly induced its expression in both undifferentiated and differentiated cells (Fig. 4c). Accordingly, we stably silenced SNAIL level in RH41 cells by transduction of RH41 cells with shRNA vectors (shSNAIL cells) and protein downregulation was verified (Supplementary Figure 2a). In those cells MYF5 expression was also induced (Fig. 4d). Moreover, temporal silencing of SNAIL expression for three days was sufficient for the induction of MYF5 expression in different ARMS cell lines: RH30 and RH41 (Fig. 4e). Interestingly, transfection of RH30 cells with the miR-30a precursor, a known negative regulator of SNAIL protein expression^18^, resulted in the downregulation of SNAIL and the upregulation of MYF5 levels (Fig. 4f). Thus, our data suggest that SNAIL is a crucial regulator of MYF5 expression in ARMS.

**Fig. 5:**
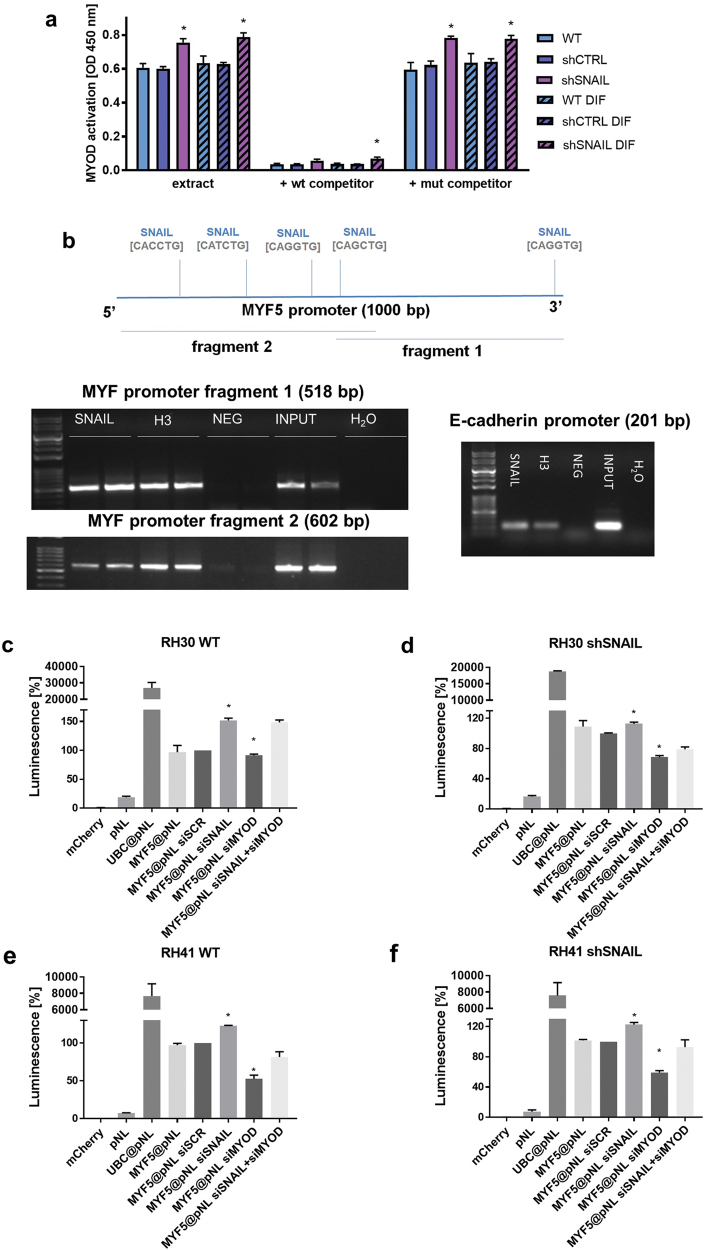
SNAIL is a direct transcriptional repressor of MYF5 expression and regulator of MYOD activity. **a** SNAIL silencing in RH30 cells induces MYOD binding to GC rich E-Box sequences. Nuclear protein extracts from both undifferentiated RH30 cells and RH30 cells differentiated for 7 days in medium supplemented with 2% HS were used in TransAM MyoD DNA-binding ELISA. To monitor the specificity of the assay, the wild-type consensus oligonucleotide (wt) and mutated (mut) sequences were used as competitors for MYOD binding from cell extracts; *n* = 3. **b** SNAIL binds to the MYF5 and E-cadherin promoters in RH30 cells. The promoter of MYF5 (~1000 bb) was screened for putative SNAIL transcription factor binding sites and the results were validated by Chip Assay. The images depict one representative result of the ChIP assay. Proteins bound to DNA were immunoprecipitated with the anti-SNAIL antibody, negative IgG control, positive histone H3 control and input DNA control was analyzed. Fragments of the MYF5 and E-cadherin promoters were amplified by PCR and visualized on agarose gels stained with ethidium bromide. **c** SNAIL silencing by siRNA in RH30 WT cells induces MYF5 promoter activation and luciferase expression when cells are transfected with MYF5@pNL plasmid (luciferase under control with MYF5 promoter), whereas MYOD silencing by siRNA does not exert any effect. SNAIL and MYOD compete for binding to MYF5 promoter in RH30 shSNAIL cells (**d**), RH41 WT (**e**), and RH41 shSNAIL cells (**f**), when cells are transfected with MYF5@pNL plasmid and siRNA against SNAIL, MYOD or siSCR (scrambled siRNA). The data were normalized to mCherry fluorescence level in each well. pNL (luciferase plasmid without promoter) served as a negative control and Ubc@pNL plasmid (luciferase under control of ubiquitin C promoter) was a positive control. The data represent the mean ± SEM or are representative images of at least 3 independent experiments. *p < 0.05

In murine myoblasts, the Snail-Hdac1/2 repressive complex binds and excludes MyoD from its targets, thus preventing MyoD occupancy on differentiation-specific regulatory elements^18^. Thus, we hypothesized that in human ARMS SNAIL may also compete with MYOD for binding to G/C rich E-Box sequences. Indeed, SNAIL silencing in RH30 cells resulted in increased MYOD binding to G/C rich E-box sequences (5′-CACCTG-3′) (Fig. 5a), indicating that SNAIL may affect the expression of MYOD-dependent targets.

Bioinformatical analysis revealed that the MYF5 promoter contains several E-box sequences (CANNTG) 1000 bp before the transcription initiation site and four of them were G/C rich E-boxes. Using the chromatin immunoprecipitation assay, we demonstrated for the first time that SNAIL binds to the MYF5 promoter in a same way that it binds to E-cadherin promoter (Fig. 5b). Those results were additionally verified using luciferase constructs under the control of MYF5 promoter (1000 bp). The plasmid with UBC promoter was used as a positive control and plasmid with luciferase without eukaryotic promoter (pNL) and mCherry plasmid were used as negative controls. Both RH30 and RH41 cells were transfected with plasmids and siRNA against SNAIL (siSNAIL), MYOD (siMYOD) and scrambled siRNA (siSCR). In RH30 WT cells SNAIL silencing by siRNA resulted in activation of MYF5 promoter, whereas MYOD silencing did not exert any effect (Fig. 5c). Those results indicated that SNAIL is a crucial repressor of MYF5 protein expression. SNAIL silencing by shRNA activated MYF5 protein expression (Fig. 4b). When RH30 shSNAIL (Fig. 5d), RH41 WT (Fig. 5e), and RH41 shSNAIL (Fig. 5f) cells were transfected with plasmids encoding luciferase under the control of MYF5 promoter, further SNAIL silencing with siRNA resulted in activation of MYF5 promoter, whereas MYOD silencing repressed it. Moreover, silencing of both MYOD and SNAIL reversed the effects (Fig. 5d–f). Those results suggested that there is a competition between SNAIL and MYOD for binding to G/C rich E-boxes in MYF5 promoter.

### SNAIL regulates expression of HDAC1/2 and forms complexes with them in ARMS

**Fig. 6:**
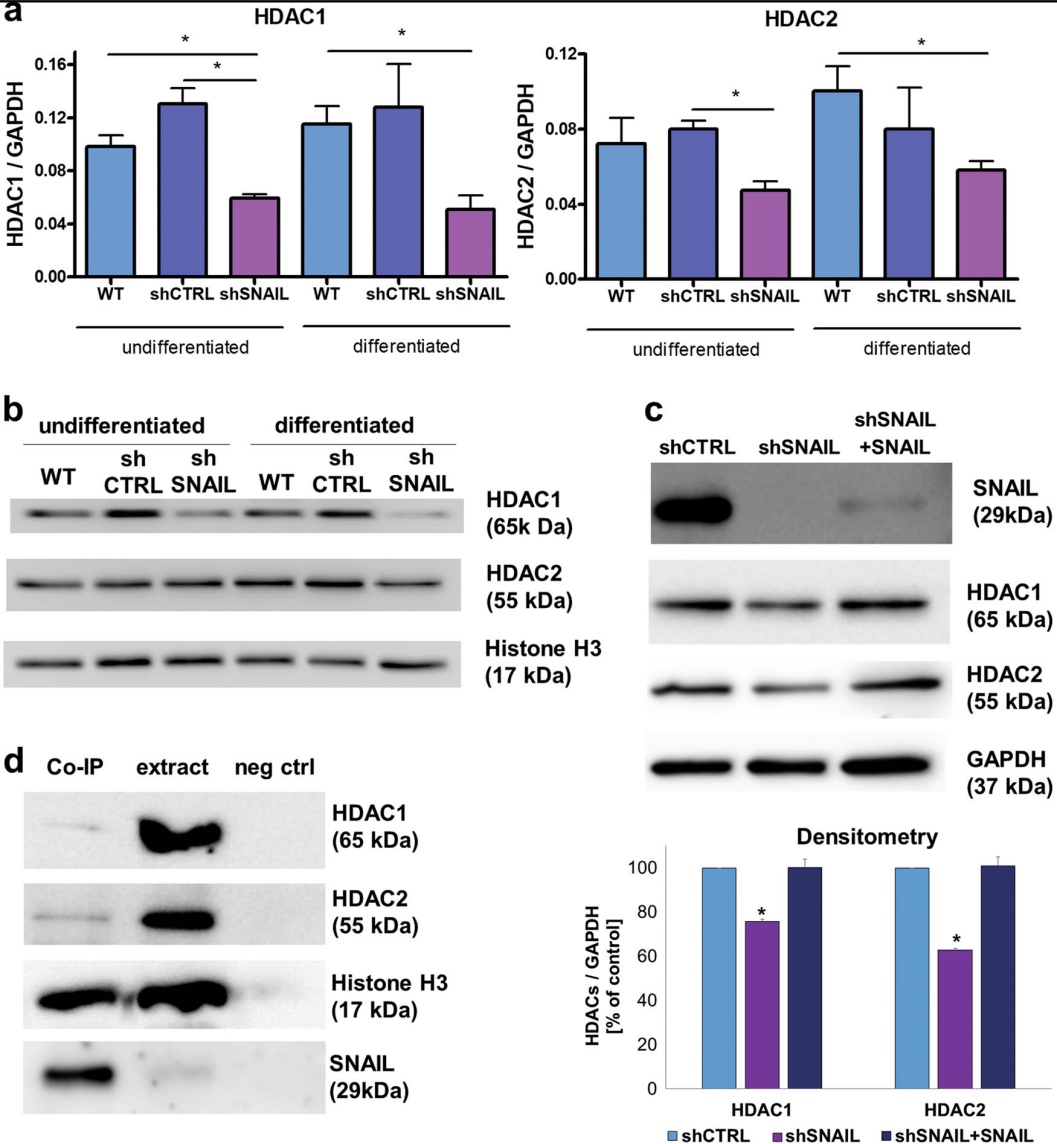
SNAIL regulates expression of HDAC1/2 and form complexes with them in ARMS. **a** HDAC1 and HDAC2 mRNA (qPCR, expression relative to GAPDH, *n* = 3) and protein (**b**) (Western blot) expression is inhibited in RH30 cells differentiated for 7 days in medium with 2% HS. **c** Transduction of RH30 shSNAIL with lentiviral vectors encoding SNAIL reverses the effect of SNAIL silencing on SNAIL, HDAC1, and HDAC2 protein levels, what was additionally confirmed by densitometric analysis (*n* = 2). **d** SNAIL form complexes with HDAC1, HDAC2 and histone H3. Co-IP of SNAIL and HDAC1, HDAC2, MYOD, histone H3 was performed. No antibody added in IP served as a negative control (neg-ctrl), whereas the input protein extract served as a positive control. The data represent the mean ± SEM or are representative images of at least 3 independent experiments. *p < 0.05

HDAC1 and HDAC2 are the components of the repressive SNAIL complex known from the literature^14^. HDAC1 was strongly and HDAC2 was slightly downregulated in differentiated RH30 shSNAIL cells both at mRNA (Fig. 6a) and at protein level (Fig. 6b). To confirm that the observed effects are SNAIL dependent, we transduced RH30 shSNAIL with lentiviral vectors encoding GFP-P2A-SNAIL, resulting in the partial restoration of SNAIL protein expression and reversal of the effect on HDAC1 and HDAC2 expression (Fig. 6c). To evaluate if SNAIL forms repressive complexes with HDAC1/2 we performed co-immunoprecipatation assay. Nuclear extract of protein was immunoprecipitated with anti-SNAIL antibody. We demonstrated that SNAIL formed indeed the complexes with HDAC1, HDAC2 and histone H3 (Fig. 6d). Those results suggest that SNAIL-dependent repression of E-box sequences is dependent on interaction of SNAIL protein with HDAC1/2.

### SNAIL silencing in ARMS induces expression of myogenic factors and muscle-specific microRNAs

**Fig. 7:**
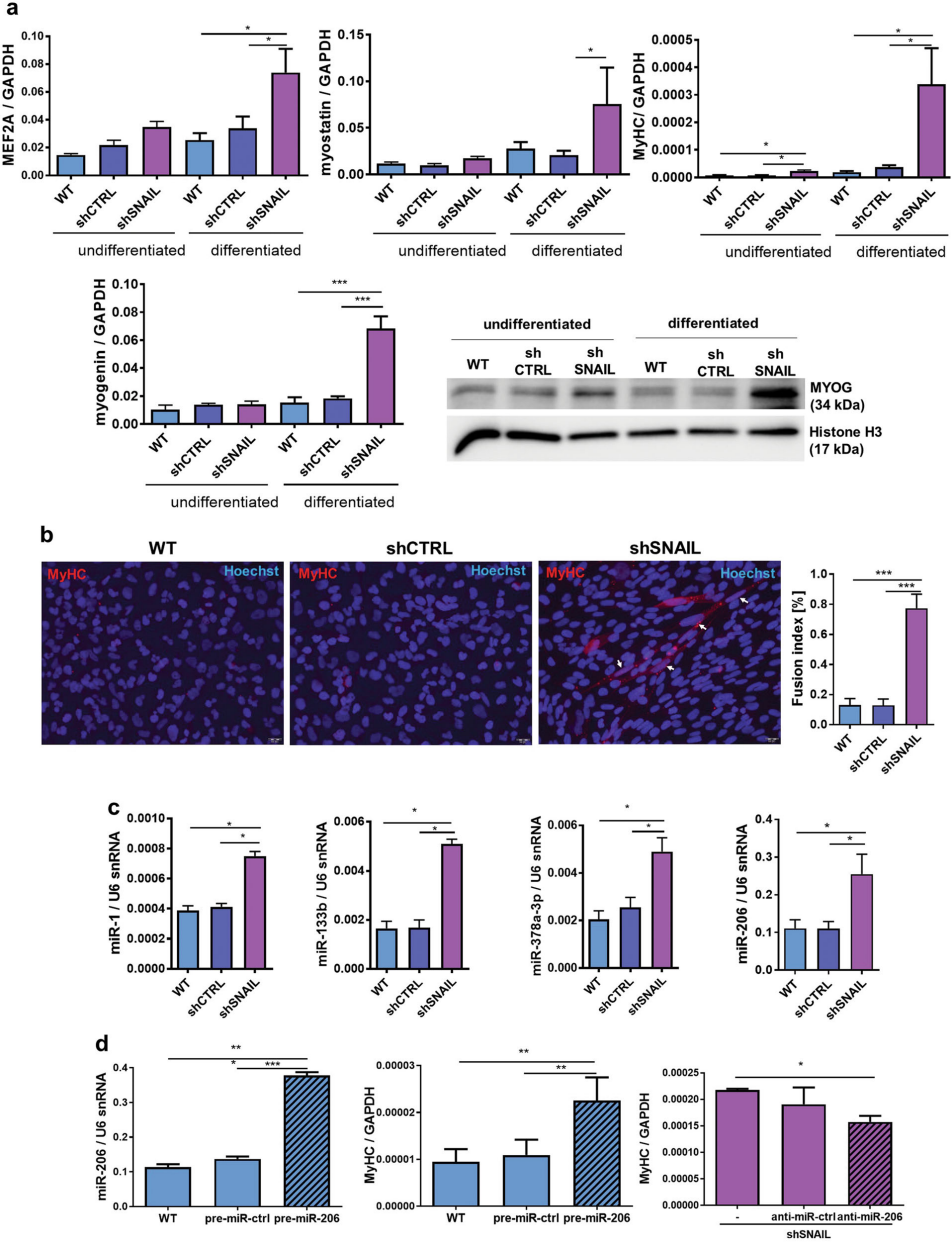
SNAIL silencing in ARMS regulates the expression of myogenic factors and microRNAs. **a** MEF2A, myogenin, myostatin and myosin heavy chain (MyHC) expression was determined by qPCR using the ΔCT quantification method and GAPDH as housekeeping gene control in undifferentiated RH30 cells or RH30 differentiated for 7 days in medium with 2% HS. SNAIL silencing and differentiation induce the expression of MEF2A, myogenin, myostatin, MyHC; *n* = 3. Myogenin (MYOG) protein was additionally verified by Western blot analysis of nuclear extracts. **b** SNAIL silencing increases the number of the spindle-shaped cells with high MyHC expression and induces their fusion. The images represent representative merged images of immunofluorescent staining for MyHC (MyHC: red; nuclei: Hoechst, blue) of RH30 cells differentiated for 8 days in medium with 2% HS. The scale bar represents 20 μm. Fusion index was calculated by expressing the number of nuclei within MyHC-positive cells with ≥2 nuclei as a percentage of the total nuclei; *n* = 4. **c** SNAIL silencing in RH30 cells upregulates expression of myomiRs, such as miR-1, miR-133b, miR-378a-3p and miR-206. The results were normalized to U6 snRNA expression level, *n* = 3. **d** Transfection of RH30 WT cells with pre-miR-206 induces the MyHC level, whereas transfection of RH30 shSNAIL cells with anti-miR-206 partially restores the effect of SNAIL silencing on the MyHC level. RH30 cells were transfected with the miRNA precursor pre-miR-206 and pre-miR negative control (pre-miR-ctrl), whereas RH30 shSNAIL cells were transfected with miRNA inhibitors: anti-miR-206 or the anti-miR negative control (anti-miR-ctrl). Twenty-four hours after transfection, the cells were treated with differentiating medium containing 2% HS for the following 48 h. miRNA expression relative to U6 snRNA and MyHC expression relative to GAPDH were validated by qPCR (qPCR, *n* = 3–4). The data in the graphs represent the mean ± SEM or are representative images of at least 3 independent experiments. *p < 0.05, **p < 0.01, ***p < 0.001

SNAIL silencing by shRNA in RH30 cells also increased the expression of other myogenic factors, including MYOD dependent or independent ones, such as MEF2A (myocyte enhancer factor 2A), myogenin, myostatin, MyHC (myosin heavy chain) at mRNA level (Fig. 7a). Moreover, our study revealed an increased expression of myogenin (Fig. 7a) and MyHC (Fig. 7b) at protein levels in RH30 shSNAIL cells . SNAIL downregulation resulted in appearance of the elongated cells with high MyHC level with more than one nuclei resembling myotubes and induced fusion of the cells (Fig. 7b). Muscle-specific microRNAs, such as miR-1, miR-133b, miR-378a-3p, and miR-206 were also upregulated in RH30 shSNAIL cells (Fig. 7c). To verify if miR-206 is a mediator of SNAIL action on MyHC level, RH30 WT cells were transfected with miR-206 precursor (pre-miR-206) and RH30 shSNAIL cells were transfected with miR-206 inhibitor (anti-miR-206). Transfection of RH30 WT cells with the miR-206 precursor increased MyHC levels, whereas inhibition of miR-206 with anti-miR sequences in RH30 shSNAIL cells partially reversed the effect of SNAIL on MyHC (Fig. 7d). Those results indicated that miR-206 is a mediator of SNAIL action on MyHC level.

By transfection with CRISPR/Cas9 nucleases targeting SNAIL we developed two ARMS cell lines—RH41 and RH30 with SNAIL knockout (Supplementary Figure 2b and 2c). By homologous directed repair of the site-specific Cas9-induced double strand brake within SNAIL gene puromycin resistance gene and red fluorescent protein (RFP) were incorporated into that locus. Cells were selected in puromycin and subsequently sorted for the brightest ones, what resulted in around 95% of the cells positive for RFP (Supplementary Figure 2d and e). Nevertheless, after 4 weeks of culture only 75% of the RH41 cells were positive for RFP and after 6 weeks of culture the population diminished to 60% (Supplementary Figure 2f). Similarly, after 4 weeks of culture the population of RFP positive RH30 cells diminished from 95 to 4% and almost disappeared after 6 weeks (Supplementary Figure 2g). Those results from RH30 and RH41 cells suggested that SNAIL knockout on both alleles may be a lethal mutation and those cells are not able to survive *in vitro*. Previously, we observed lethality of RH30 cells with diminished SNAIL level after shRNA *in vivo*.

When we characterized those cell lines we observed that differentiating RH41 SNAIL deficient cells acquired spindle-shaped morphology (Supplementary Figure 2h). RH30 CRISPR cells became elongated even in standard culture conditions (Supplementary Figure 2j). An increased expression of myogenic factors, such as MEF2A, myogenin, myostatin and MyHC was also observed (Supplementary Figure 2i and 2b).

**Fig. 8:**
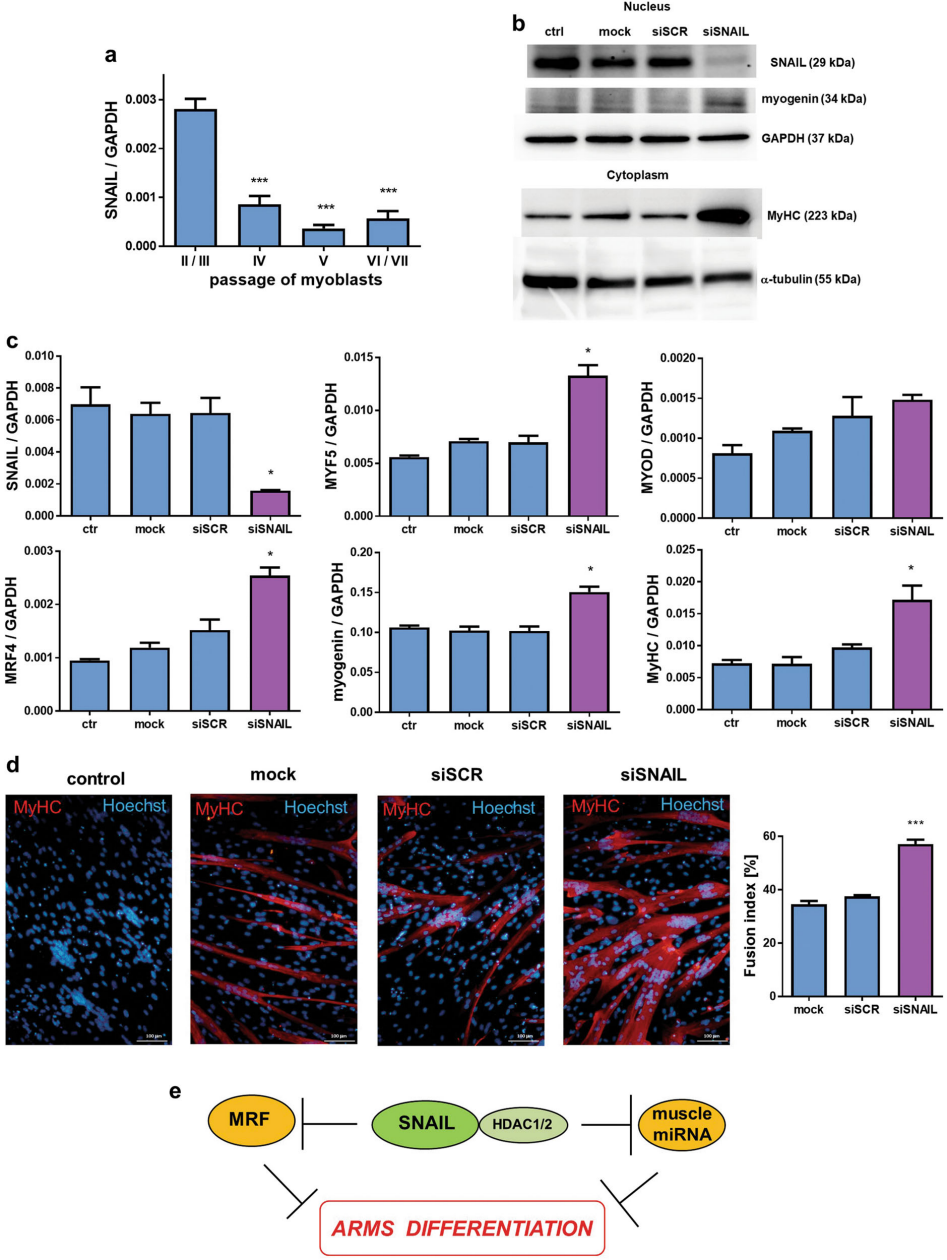
SNAIL silencing in human myoblasts increases expression of myogenic factors. **a** SNAIL expression in human myoblasts diminishes with subsequent passages (qPCR, *n* = 4). **b** Human myoblasts were transfected with siRNA against SNAIL (siSNAIL) and with a scrambled siRNA sequence (siRNA) or they were treated with transfection reagent (mock). 24 h after transfection they were treated with a differentiating medium containing 2% HS for the next 48 h. SNAIL silencing was validated by Western blot. SNAIL silencing increased the myogenin and MyHC protein levels (Western blot). **c** Expression of SNAIL, MYF5, MYOD, MRF4, myogenin and MyHC was determined by qPCR with ΔCT quantification method and GAPDH as a housekeeping gene control. SNAIL silencing and differentiation induces expression of the analyzed myogenic factors. **d** SNAIL silencing increases the number of the myotubes with high MyHC expression and induces their fusion. The images are representative merged images of immunofluorescent staining for MyHC (MyHC: red; nuclei: Hoechst, blue) of myotubes. The scale bar represents 100 μm. Fusion index was calculated by expressing the number of nuclei within MyHC-positive myotubes with ≥2 nuclei as a percentage of the total nuclei; *n* = 4. **e** Mechanism of action of SNAIL transcription factor on myogenic differentiation of ARMS. Data on graphs are represented as mean ± SEM. *p < 0.05, *n* = 4

These data suggest that we discovered a novel non-canonical mechanism of SNAIL action, which may be of significance in the regulation of human physiological and pathological myogenesis. Interestingly, when human primary myoblasts were cultured for several passages, what led to their differentiation, the SNAIL level decreased (Fig. 8a). Accordingly, the transient downregulation of SNAIL levels by transfection with siRNA resulted in the increased myoblasts differentiation and upregulation of early and late differentiation factors, such as MYF5, MRF4, myogenin and MyHC at mRNA or protein level (Fig. 8b, c), suggesting that SNAIL plays a crucial role in the regulation of myogenic differentiation. Indeed, SNAIL silencing increased fusion of the cells and appearance of multinucleated myotubes (Fig. 8d).

These results indicate for the first time that muscle-specific miRNAs and myogenic regulatory factors are important mediators of SNAIL anti-myogenic action in ARMS (Fig. 8e).

## Discussion

This study was undertaken to investigate the novel role of the SNAIL transcription factor in the regulation of ARMS growth and myogenic differentiation. SNAIL is known as a master regulator of the growth and metastasis of epithelial tumors, and its canonical action involves the induction of the EMT^11^. Interestingly, the role of SNAIL in the biology of mesenchymal tumors is poorly understood. Here, we showed that SNAIL regulates differentiation of ARMS cells. This is exerted through regulation of myogenic factors and microRNAs. We revealed the novel mechanism of the anti-myogenic action of human SNAIL, a function that may be relevant not only in RMS but also in other physiological processes.

In our studies, we demonstrated for the first time that SNAIL is a crucial negative regulator of myogenic differentiation in ARMS through disruption of early myogenic transcription factors functions; therefore, SNAIL may be a novel target for future therapies. We demonstrated also that SNAIL is crucial for the differentiation of human primary myoblasts. Our results are supported by the recent papers demonstrating that, Snail regulates the differentiation of murine myoblasts. Inhibition of Snail leads to the induction of myogenic differentiation of these cells^18^, whereas its upregulation inhibits C2C12 myotube differentiation^22^. Additionally, a recent study demonstrated that murine Snail also controls cardiomyocyte development in embryogenesis and that its expression is important in mesodermal commitment and differentiation^23^.

RMS cells display expression of several myogenic factors, but they do not complete normal myogenic differentiation^1^. We suggest that SNAIL may be a crucial factor responsible for the dysregulation of myogenic differentiation, leading to ARMS development. Because ERMS may be derived from impaired differentiation of MSCs, in our previous studies we demonstrated that the constitutive activation of MET receptor signaling in MSCs may lead to their myogenic differentiation probably by regulation of SNAIL expression^4,24^. In the current study, we describe the novel mechanism of SNAIL action in ARMS and demonstrate that SNAIL regulates two important early myogenic factors in RMS.

SNAIL decreases MYOD binding to G/C-rich E-boxes, with possible implications for the physiological myogenesis. Early efforts to investigate the phenomenon of the impaired differentiation of RMS focused on MYOD. Previous papers have suggested that RMS cells express MYOD, but it appears to be nonfunctional as a transcriptional coactivator^25^. Based on our data, SNAIL appears to be a novel factor that regulates MYOD transcriptional activity, a finding that has been lacking previously. In murine myoblasts, inhibition of the Snail complex with Hdac1/2 leads to the induction of myogenic differentiation by competition with MyoD for binding to E-box sequences^18^. Our studies suggest that a similar mechanism of action is also crucial for the myogenic differentiation of human RMS cells. SNAIL mediates the repression of different gene promoters by recruitment of the HDAC1/HDAC2 complex^14^. We showed that in differentiating ARMS cells, SNAIL recruits and regulates the expression of HDAC1 and HDAC2.

Importantly, in RMS, the SNAIL transcription factor is an important regulator of the MYOD-miR-206-MyHC axis, thereby regulating myogenic differentiation. MYOD induces the expression of numerous myogenic genes, such as desmin, MEF2A and myogenin, that are regulated by Snail and Slug in murine myoblasts^18^. One of the downstream mediators of MYOD is miR-206 (ref. ^8^), which induces the late myogenic differentiation of RMS cells and the expression of myosin heavy chain (MyHC). miR-206 inhibits RMS growth by the induction of differentiation^26^, and miR-206 induces the expression of MyHC-positive differentiating myoblasts^27^.

Both MYF5 and MYOD were demonstrated to be important transcriptional targets regulating human RMS growth^28^. In early development both factors are required for the formation of skeletal myoblasts, and mutant mice without both factors do not develop skeletal muscles^29^. Muscle progenitors with MYOD expression but lacking MYF5 expression may either remain highly proliferative and developmentally arrested or differentiate into mesenchymal progeny^30^, suggesting that MYOD is a crucial regulator of the proliferation to differentiation switch. Determination of the myogenic fate of stem cells requires the progressive expression of MYF5, MYOD, and subsequently myogenin (MYOG) and myofibrillar proteins, such as the myosin heavy chain. Progenitors expressing MYF5 and MYOD can divide several times before terminal differentiation^31^. Accordingly, RH30 ARMS cells with silenced SNAIL are MYF5 and MYOD positive. These cells divide *in vitro* but do not form any tumors *in vivo*.

Importantly, our data demonstrated that SNAIL negatively regulates MYF5 expression in ARMS. These data are consistent with those of previous studies indicating that MYF5 expression is increased in ERMS cells compared with more proliferative ARMS cells. Those studies also suggested that MYF5 is a novel RMS class predictor^32^. Additionally, ERMS cells positive for MYF5 do not display pro-invasive capabilities, but they play supportive roles in tumor progression^33^. Accordingly, PAX3/7-FOXO1 translocation-positive ARMS cells do not display MYF5 expression^32^. In this study through SNAIL silencing we demonstrated for the first time that SNAIL is the main inhibitor of MYF5 expression in ARMS displaying PAX3-FOXO1 translocation. PAX3-FOXO1 translocation is present in RH30 and RH41 cells, and it cannot be excluded that PAX3-FOXO1 may act upstream of SNAIL in the regulation of MYF5 expression. In mice, Myf5 gene contains a large regulatory region 145 kb 5′ upstream of the transcription start site. Numerous regulatory transcription factors can bind to this region, including Pax3, which binds to the −58/−56 kb distal Myf5 enhancer^34^. Accordingly, in RMS, PAX3-FOXO1 also binds to a similar region^35^. Importantly, PAX3 is an upstream regulator of SNAIL and SLUG in embryonic development^36^. Therefore, in the future, the effect of PAX3-FOXO1 on SNAIL activity should also be investigated, as SNAIL level may be elevated in ARMS compared to ERMS due to PAX3-FOXO1 expression in ARMS.

Interestingly, approximately 10% of satellite cells in quiescent muscles never express MYF5. These cells display self-renewal potential^37^, suggesting that the induction of MYF5 expression induces subsequent differentiation steps. The regulation of the MYF5 level appears to be crucial in ARMS growth, and MYF5 may be one of the most important mediators of SNAIL action. We demonstrated a novel mechanism for SNAIL: it binds directly to the MYF5 promoter and inhibits its expression in ARMS tumors, thereby regulating myogenic differentiation. Surprisingly, in chick embryos SNAIL was demonstrated to be a necessary and sufficient step for the NOTCH-dependent activation of MYF5 in the medial border of the dermomyotome^38^, what suggests either its opposite role in different species or in normal myogenesis.

In conclusion, the induction of ARMS differentiation by SNAIL silencing leads to the complete inhibition of tumor growth, suggesting that SNAIL may be a novel target for differentiation-based therapies in human ARMS. The mechanism of SNAIL action on MYF5 expression and MYOD activity may also be of significance for further studies on myogenesis during embryonic development.

## Materials and methods

### Cell culture

RMS cell lines (RH30, RH41, RD) were kindly provided by Dr. PJ Houghton (Center for Childhood Cancer, Columbus, OH, USA) or ordered from the American Type Culture Collection (ATCC, Manassas, VA, USA). RH18 ERMS cell line was ordered from DSMZ (Leibniz Institute DSMZ-German Collection of Microorganisms and Cell Cultures in Germany). The cells were cultured in DMEM high-glucose medium (Lonza Group Ltd., Basel, Switzerland) supplemented with 10% fetal bovine serum (FBS, EURx, Gdansk, Poland) and 50 μg/ml gentamicin (Lonza) at 37 °C, 5% CO_2_ and 95% humidity. The RMS cell lines were differentiated in DMEM low-glucose medium (Lonza) supplemented with 2% horse serum (HS) (Gibco, BRL Grand Island, NY, USA). At least 7 days of incubation in differentiating medium was required to observe single fusing cells. Shorter incubation periods resulted only in strong elongation of the cells (acquisition of spindle shape). The cellular morphology was visualized using Wright’s stain (Sigma-Aldrich, St. Louis, MO, USA).

The cell lines were routinely tested for *Mycoplasma* contamination using by MycoAlert™ Mycoplasma Detection Kit (Lonza). Cell line authentication was performed by STR profiling using AmpFlSTR SGM PLUS Kit (Applied Biosystems, Foster City, CA, USA) and sequencing apparatus ABI Prism 310 Genetic Analyser (Applied Biosystems) according to the manufacturer’s protocol.

Primary human myoblasts were isolated by our lab and characterized as previously described^39^. These cells were cultured in DMEM/F12 medium (Lonza) supplemented with dexamethasone, insulin (both from Sigma-Aldrich) 18% FBS (EURx), EGF (R&D Systems, Minneapolis, MN, USA), FGF (R&D), HGF (R&D) and gentamicin (Lonza). They were differentiated in DMEM low-glucose medium (Lonza) supplemented with 2% horse serum (HS) (Gibco).

### Production of viral vectors and transduction of cells

RH30 and RH41 cells were transduced with shRNA Lentiviral Particles targeting SNAIL and control lentiviral particles at an MOI of 2.5 (Santa Cruz Biotechnology, Santa Cruz, CA, USA; sc-38398-V and sc-108080) in 6 μg/ml polybrene (Sigma, St. Louis, MO, USA) according to the manufacturer’s protocol. SNAIL shRNA lentiviral particles are a pool of concentrated, transduction-ready viral particles containing 3 different target–specific constructs that encode 19–25 nt (plus hairpin) shRNA designed to knock down SNAIL. Transduced cells were selected with 0.5 μg/ml puromycin (InvivoGen, San Diego, CA, USA). Lentiviral particles encoding GFP-P2A-SNAIL (GFP-P2A-SNAIL @pLenti6/UbC) were produced using the Vira Power Lentiviral Expression System (Invitrogen, Carlsbad, CA, USA), as previously described^40^. RH30 shSNAIL cells were transduced with GFP-P2A-SNAIL lentiviral vectors (at MOI = 10) in the presence of 6 μg/ml polybrene (Sigma-Aldrich). After 72 h the cells were subject to selection with 5 μg/ml blasticidin (InvivoGen) for 2 weeks.

### Transfection with siRNA

RH30, RH41 cells and human myoblasts were transfected with 20 nM siRNA against SNAIL (combination of two Silencer Select siRNA ID variants: s13185 and s13187, Ambion Inc., Austin, TX, USA) or scrambled control siRNA (Silencer Select Negative Control #1 siRNA, cat. 4390844, Ambion) using Lipofectamine 2000 (Invitrogen) or Lipofectamine RNAiMAX transfection reagent according to vendor’s instructions. Twenty-four hours later, the transduction medium was changed to differentiating medium supplemented with 2% HS. RNA or protein was isolated 72 h after transfection. The cellular morphology was visualized using Wright’s stain (Sigma-Aldrich). Proliferation of RH30 and RH41 cells transfected on 96-well plates with siRNA was estimated using CellTiter 96® AQueous One Solution assay (Promega, WI, USA), according to vendor’s protocol.

### Transfection of cells with miRNA precursors and inhibitors

RH30 cells were transfected with 30 nM pre-miR-30a-5p (ID: PM11062, Ambion) or 30 nM pre-miR-206 (ID: PM10409, Ambion) miRNA precursors and pre-miR negative controls (ID: AM17110, Ambion) or alternatively with 30 nM anti-miR miRNA inhibitors against miR-206 (ID: AM10409, Ambion) and negative controls (ID: AM17010, Ambion) using the siPORT NeoFX transfection reagent (Ambion) according to the manufacturer’s instructions, as described previously^41^. Twenty-four hours later, the transduction medium was changed to differentiating medium supplemented with 2% HS. RNA was isolated 72 h after transfection.

### Generation of SNAIL CRISPR knockout

8 × 10^4^ RH30 and RH41 cells were seeded per one well of 24-well plate. The next day the cells were transfected with 500 ng of SNAIL CRISPR/Cas9 KO plasmid (Santa Cruz Biotechnology, sc-400244) and 500 ng of SNAIL HDR plasmid (Santa Cruz Biotechnology, sc-400244-HDR) using Lipofectamine 2000 (Invitrogen) according to vendor’s instructions. SNAIL CRISPR/Cas9 KO plasmid consists of a pool of 3 plasmids, each encoding the Cas9 nuclease and a target-specific 20 nt guide RNA designed for maximum knockout efficiency. SNAIL HDR plasmid consists of a pool of 2–3 plasmids, each containing a homology directed DNA repair (HDR) templates corresponding to the cut sites generated by the SNAIL CRISPR/Cas9 KO plasmid. Each HDR template contains two 800 bp homology arms designed to specifically bind to the genomic DNA surrounding the corresponding Cas9-induced double strand DNA break site. During the repair, the SNAIL HDR plasmid incorporates a puromycin resistance gene and red fluorescence gene (RFP). Seventy-two hours after transfection, the RH30 and RH41 cells were selected with 0.5 μg/ml puromycin (InvivoGen, San Diego, CA, USA) for 2 weeks and subsequently they were sorted to select the cells with the brightest expression of RFP using BD FACSAria (BD Biosciences). Successful co-transfection of the CRISPR/Cas9 KO Plasmid and HDR Plasmid was visually confirmed by detection of RFP *via* fluorescent microscopy and using Attune flow cytometer (ThermoFisher Scientific). Around 95% of the cells were positive for RFP after the sorting.

### Bioinformatical analysis of microarray data from RMS patients

For gene expression analysis in a group of 158 RMS patients we used data from GEO database, stored under accession number GSE92689 (ref. ^20^). Background subtraction and data normalization was performed with affy package[21] in R/Bioconductor and the average expression was used for further statistical analysis. Pearson correlation of gene expression was analyzed using GraphPad Prism software. Statistical analysis of SNAIL expression in RMS stages and RMS subtypes was performed by Mann–Whitney test using GraphPad Prism software.

### DNA and RNA isolation and reverse transcription

Total RNA was extracted using the Universal RNA purification kit (EURx), according to the manufacturer’s protocol. For the analysis of both miRNA and mRNA expression, total RNA was isolated using the mirVana miRNA Isolation Kit (Ambion). Reverse transcription of mRNA was performed using MMLV reverse transcriptase (Promega, Madison, WI, USA) according to the manufacturer’s protocol. Reverse transcription of both miRNA and mRNA was performed using the NCode VILO miRNA cDNA Synthesis Kit (Invitrogen), according to the manufacturer’s protocol.

### Quantitative real-time PCR

Gene expression was determined by qRT-PCR analysis using ABI PRISM 7300 Sequence Detection System or Quant Studio 7 Flex System (both from Applied Biosystems, Foster City, CA, USA), Blank qPCR Master Mix (EURx) and the indicated Taq-Man probes (Applied Biosystems): human: GAPDH (Hs99999905_m1), MYF5 (Hs00271574_m1), MYOD (Hs00159528_m1), MRF4 (Hs01547104_g1), MEF2A (Hs01050409_m1), MYOSTATIN (Hs00976237_m1), MYOGENIN (Hs01032275_m1), MYH2 (Hs00430042_m1), SNAI1 (Hs00195591_m1), HDAC1 (Hs02621185_s1) and HDAC2 (Hs00231032_m1). The mRNA expression level for all of the samples was normalized to the housekeeping gene GAPDH (Hs99999905_m1), using the 2^−ΔCt^ method.

For the evaluation of miRNA expression by quantitative real-time PCR, SYBR Green qPCR Master Mix (EURx) and universal reverse primer from the NCode VILO miRNA cDNA Synthesis Kit (Invitrogen) were used with the indicated forward primers. The miRNAs expression levels were quantified using the 2^−ΔCt^ method, using U6 snRNA as a relative control.

U6 snRNA: 5′-CGCAAGGATGACACGCAAATTC-3′

miR-1: 5′-GCTGGAATGTAAAGAAGTATGTATAA 3'

miR-133b: 5′-TTTGGTCCCCTTCAACCAGCTA-3′

miR-206: 5′-TGGAATGTAAGGAAGTGTGTGG-3′

miR-378a-3p: 5′-ACTGGACTTGGAGTCAGAAGG-3′

### Western blot

Protein (either nuclear and cytoplasmic fractions or total extracts) was isolated using the Nuclear Extract Kit (Active Motif, La Hulpe Belgium) according to the manufacturer’s protocol. The protein concentration was measured using the Bio-Rad Protein Assay (Bio-Rad, Hercules, California, USA) according to the manufacturer’s protocol. Western blot was performed using the anti-GAPDH rabbit mAb (14C10; Cell Signaling Technology, Leiden, The Netherlands), the anti-α-tubulin mAb (DM1A; Calbiochem, EMD Chemicals Inc San Diego, USA), the anti-histone H3 (ab1791; Abcam, Cambridge UK), the anti-SNAIL mouse mAb (L70G2; Cell Signaling), the anti-MYOD rabbit pAb (M-318; Santa Cruz, CA, USA), the anti-HDAC1 rabbit pAb (Poly6074 clone; Biolegend, San Diego CA, USA), the anti-HDAC2 mouse mAb (Biolegend, 3F3/HDAC2), anti-myogenin mouse mAb (F5D, sc-12732, Santa Cruz Biotechnology), anti-fast myosin skeletal heavy chain mouse mAb antibody (MyHC, MY-32, ab51263, Abcam), and secondary anti-rabbit and anti-mouse antibodies conjugated with horseradish peroxidase (HRP, Santa Cruz Biotechnology) as previously described^24^.

### Transcriptional activity studies

The activation of MYOD transcription factor and its binding to E-Box sequences were evaluated using isolated nuclear protein extracts from RH30 cells and TransAM® MyoD DNA-binding ELISA enzyme-linked immunosorbent assay (ELISA; Active Motif) for activated MYOD transcription factor according to the manufacturer’s protocol and using proteins from nuclear extracts. To monitor the specificity of the assay the WT consensus oligonucleotide and mutated sequences were used as competitors for MYOD binding from cell extracts.

### Bioinformatic analysis

The promoter of MYF5 (~1000 bb) was screened for putative SNAIL transcription factor binding sites using a TF prediction tool called ConSite (http://consite.genereg.net/)^42^. The results were then compared with other TF prediction tools.

### Chromatin immunoprecipitation (ChIP) assay

ChIP was performed using SimpleChip Enzymatic Chromatin IP Kit (Cell Signaling Technology) according to the manufacturer’s protocol. For ChIP assays 10 µg of antibodies against SNAIL (SNAI1 E-18: sc10432X, Santa Cruz Biotechnology), 10 µg of the positive control histone H3 (D2B12 XP Rabbit mAb Chip formulated, Cell Signaling Technology) and 1 µg of the negative control IgG from the kit were used. After immunoprecipitation, the DNA was isolated using spin columns from the kit and eluted in 50 µl Elution Reagent C. PCR was performed with 2 µl of immunoprecipitated material and the products were analyzed on an 1.5% agarose gel, and visualized using a gel documentation system. The following primers were used to quantify SNAIL binding to the MYF5 promoter region 500 and 1000 bp before the translation start site and to the E-cadherin promoter (positive control for SNAIL targets).

MYF5 primer fragment 1 forward

5′-GCCAGCTGAAAGGGATTTCTATT-3′

MYF5 primer fragment 1 reverse

5′-CACGTCCATCCTGCTGAGAG-3′

MYF5 primer fragment 2 forward

5′-AAAACTGGGCTTCTTCTGTTGG-3′

MYF5 primer fragment 2 reverse

5′-GACCAAGGCAGTCCAACTTTTT-3′

ECADH promoter primer forward

5′-TAGAGGGTCACCGCGTCTAT-3′

ECADH promoter primer reverse

5′-TCACAGGTGCTTTGCAGTTC-3′

### Luciferase activity assay

NanoLuc® luciferase plasmids variants (pNL plasmids) were ordered from Promega, WI, USA. pNL plasmids with luciferase under control of human ubiquitin C (UBC@pNL) and MYF5 promoter (MYF5@pNL) were generated by us. Promoter fragments were cloned from human genomic DNA using primers:

5′-TCAACTCGAGAAAACTGGGCTTCTTCTGTTGG,

5′-TAACAAGCTTCACGTCCATCCTGCTGAGAG for MYF5 promoter

and 5′-TAATCTCGAGGATCTGGCCTCCGC,

5′- GGGGGAGATCTCTTCGTCTAACAAAAAAGCC for UBC promoter.

Primers were designed to introduce restriction sites appropriate for following ligation with linearized pNL plasmids using T4 DNA Ligase (Invitrogen, USA). Bacterial transformations of One Shot™ TOP10 Chemically Competent E. coli (Invitrogen, USA) with generated UBC@pNL and MYF5@pNL plasmids were performed and LB Agar plates with 100 µg/ml ampicillin were used for bacterial selection. 6 clones of each plasmid were purified from selected bacterial colonies using QIAfilter Plasmid Maxi Kit (Qiagen, Germany). Sequences of MYF5 and UbC promoters in pNL plasmids were confirmed with Sanger sequencing—BigDye™ Terminator v3.1 Cycle Sequencing Kit (Thermo) and Applied Biosystems 3500 Genetic Analyzer.

RH30 and RH41 cells were transfected with pNL, UBC@pNL and MYF5@pNL plasmids using Lipofectamine 2000 (Invitrogen) transfection reagent according to the manufacturer’s instructions, utilizing 500 ng of pNL plasmids, and 100 ng mCherry plasmid, and 20 nM siRNA against SNAIL (combination of two Silencer Select siRNA ID variants: s13185 and s13187, Ambion Inc., Austin, TX, USA) or siRNA against MYOD (combination of two Silencer Select siRNA ID variants: s9231 and s9229) or scrambled control siRNA (Silencer Select Negative Control #1 siRNA, cat. 4390844, Ambion) and 1.0 μl of Lipofectamine 2000 per one well of a 24-well plate. Forty-eight hours after transfection, the cells were evaluated for activity of the secreted NanoLuc® luciferase using Nano-Glo® Luciferase Assay (Promega, WI, USA) according to the vendor’s protocol. The results of luminescence were normalized to mCherry fluorescence level in each well. Luminescence and fluorescence signals were analyzed using Tecan Spark 10 M microplate reader (Tecan Trading AG, Switzerland).

### Co-immunoprecipitation

RH30 cells were grown to confluence on 100 mm plates and nuclear extracts were prepared using the Nuclear Complex Co-IP Kit (Active Motif) according to vendor’s protocol. 100 μg of nuclear extract was used per IP reaction and incubated with either 2 μg of anti-SNAIL antibody (Sc-10432, Santa Cruz, CA, USA) or no antibody using IP Low Buffer from the kit. Protein G beads were added to each IP reaction. Following the IP, 2× Laemmli Sample Buffer was added to each IP reaction, samples were boiled and run on SDS-PAGE gel. Western blot analysis was performed using the anti-HDAC1 rabbit pAb (Poly6074 clone; Biolegend, San Diego CA, USA), the anti-HDAC2 mouse mAb (Biolegend, 3F3/HDAC2), the anti-histone H3 (ab1791; Abcam, Cambridge UK), anti-SNAIL mouse mAb (L70G2; Cell Signaling) and secondary anti-rabbit and anti-mouse antibodies conjugated with horseradish peroxidase (HRP, Santa Cruz Biotechnology) as previously described. The input RH30 nuclear extract was run as a positive control for western blot.

### Cell cycle and BrdU incorporation

For the assessment of DNA content and BrdU incorporation, RH30 cells were differentiated for 7 days in DMEM medium with 2% HS and then they were analyzed using APC BrdU flow Kit (BD Pharmingen, CA, USA) using Attune flow cytometer (ThermoFisher Scientific), according to vendor’s protocol.

### Immunofluorescent staining

RH30 cells or myoblasts were fixed in 4% formaldehyde (POCH) in PBS, permeabilized in 0.1% TritonX-100 (Sigma-Aldrich), blocked in 1% bovine serum albumin (BSA, Sigma-Aldrich), incubated with rabbit anti-MYF5 monoclonal antibody (ab125078, Abcam) or anti-fast myosin skeletal heavy chain antibody (MyHC, MY-32, ab51263, Abcam), and then incubated with secondary goat anti-rabbit or anti-mouse antibodies conjugated with Alexa Fluor 555 (Life Technologies) and sometimes with Hoechst. The stained slides were mounted in Vectashield mounting medium with DAPI (Vector Laboratories Inc. Burlingame, CA, USA) or Dako Fluorescence Mounting Medium (Dako, Denmark). For visualization of morphology, the cells were stained with Wright’s dye (Sigma-Aldrich). Labeling was assessed by fluorescence microscopy using an Olympus BX51 or IX70 microscope (Olympus Corporation, Tokyo, Japan) and Olympus XC50 camera with cellSens Dimension software (both from Olympus). The images were processed using cellSens Dimension software or ImageJ software (National Institute of Health, USA). To quantify RH30 cells and myoblasts fusion, we calculated the fusion index by expressing the number of nuclei within MyHC-positive cells with ≥2 nuclei as a percentage of the total nuclei.

### *In vivo* experiments

Animal experiments were approved by the Local Ethics Committee in Krakow in Poland. In total, 5 × 10^6^ RH30 cells were injected subcutaneously into 6- to 8-week-old NOD-SCID mice. Each experimental group contained 4 to 5 animals, and all of the experiments were repeated twice. Tumor size was evaluated using a caliper. Tumor volume was estimated using the formula *V* = *D* × *d*^2^ × 0.5 (where *V* is the tumor volume, *D* is the largest dimension, and *d* is the smallest dimension). After 3 to 4 weeks, the mice were euthanized, and their tumors were harvested. After the evaluation of tumor weight, the tumor sections were fixed in formalin. The tumor sections were stained with hematoxylin-eosin to visualize tumor morphology using Dako EnVision Detection Systems (Dako Polska Sp. z o.o.), as previously described^24^.

To evaluate if treatment of growing tumors with SNAIL siRNA can affect tumor growth, 5 × 10^6^ RH30 cells were injected subcutaneously into 6- to 8-week-old NOD-SCID mice. After tumors reached an average size of 60 mm^3^, mice were injected intratumorally with PBS, scrambled siRNA (siSCR, Silencer Select Negative Control #1 siRNA, Ambion) or siRNA against SNAIL (combination of two Silencer Select siRNA ID variants: s13185 and s13187, Ambion) at dose 3 μg/kg of body weight. Tumor growth was monitored with caliper . Three injections were performed 9, 11, and 14 days after implantation of the cells. Tumor volume was evaluated with caliper until day 17 and it was calculated as percentage of volume from the start of the therapy. Weight of tumor was analyzed 17 days after the implantation. The tumor sections were stained with hematoxylin-eosin to visualize tumor morphology using Dako EnVision Detection Systems (Dako Polska Sp. z o.o.) and after deparaffinization they were stained immunohistochemically, as described previously^24^, with anti-SNAIL primary mouse monoclonal antibody to evaluate SNAIL expression (1:50; Santa Cruz Biotechnology; G-7: sc-271977). Each experimental group contained 3 mice.

### Statistical analysis

Unless stated otherwise, the results show the mean ± standard error of the mean (SEM) of at least 3 to 4 independent experiments. Statistical analysis was performed by one-way analysis of variance (ANOVA) with Tukey post-test or Student’s *t*-test using GraphPad Prism software. Differences with a *p*-value less than 0.05 were considered statistically significant. *n* value in figure legends describes the number of independent biological experiments.

## Electronic supplementary material

Supplementary Figure Legends

Supplementary Figure 1

Supplementary Figure 2
