# Supplementary material for: SNAIL is a key regulator of alveolar rhabdomyosarcoma tumor growth and differentiation through repression of MYF5 and MYOD function
Source: Cell Death Dis. 2018 May 29;9(6):643. doi: 10.1038/s41419-018-0693-8 (PMC5974324; doi:10.1038/s41419-018-0693-8)
Supplement: Supplementary file 1 — Supplementary Figure Legends [file 41419_2018_693_MOESM1_ESM.docx]

**SUPPLEMENTARY FIGURE LEGENDS**

**Supplementary Figure 1. SNAIL silencing in RH30 and RH41 ARMS cells induces a spindle shaped phenotype and inhibits proliferation.**

SNAIL silencing by two different siRNA sequence variants induces the elongation of RH41 and RH30 cells and diminishes their proliferation (Wright’s staining). RH30 ARMS cells were transfected with siRNA sequences against SNAIL (siSNAIL) and a scrambled siRNA sequence (siRNA). 24 hours after transfection, the medium was changed for a differentiating medium containing 2% HS for the next two days. Error bar represents 100 μm.

**Supplementary Figure 2. SNAIL CRISPR knockout in RH41 ARMS cells changes morphology of the cells and inhibits proliferation.**

(**a**) To stably silence the SNAIL level, RH41 cells were transduced with shRNA lentiviral vectors targeting SNAIL (shSNAIL) and control vectors (shCTRL), and these cells were selected with puromycin. SNAIL silencing was validated by Western blot (nuclear extracts). (**b**) RH41 cells with SNAIL knockout were generated by transfection with plasmid encoding CRISPR/Cas9 nucleases targeting SNAIL gene and HDR plasmid allowing incorporation of puromycin resistance gene and red fluorescent protein (RFP) into SNAIL gene locus. Subsequently the cells were selected in puromycin and sorted for the cells with the brightest RFP expression. SNAIL expression level was validated by Western blot. SNAIL deficient cells displayed also slightly higher expression of myogenin (MYOG) protein. (**c**) RH30 cells with SNAIL knockout were generated by transfection with plasmid encoding CRISPR/Cas9 nucleases targeting SNAIL gene and HDR plasmid SNAIL expression level was validated by Western blot two weeks after sorting and SNAIL protein level was compared to RH30 shSNAIL (with SNAIL level downregulated by shRNA). Next, RFP level after the sorting was validated by florescent microscopy in RH41 CRISPR (**d**) and RH30 CRISPR (**e**) cells. Around 95% of the cells displayed RFP expression (scale bar represents 100 μm). (**f**) RFP incorporated into SNAIL gene locus in RH41 cells diminished from around 95% after the cell sorting to 75% after 4 weeks and to 60% after 6 weeks. (**g**) RFP incorporated into SNAIL gene locus in RH30 cells diminished from around 95% after the cell sorting to 4% after 4 weeks and almost disappeared after 6 weeks, suggesting that SNAIL knockout cells undergo cell death in culture *in vitro*. (**h)** SNAIL CRISPR knockout RH41 cells displayed a spindle shape morphology in a differentiating medium with 2% HS for 6 days (error bar represents 100 μm). (**j)** SNAIL CRISPR knockout RH30 cells displayed a spindle shape morphology in standard culture conditions (error bar represents 100 μm). (**i**) SNAIL CRISPR knockout cells displayed an increased expression of MEF2A, myogenin, myostatin and myosin heavy chain (MyHC) (qPCR using the ΔCT quantification method and GAPDH as housekeeping gene control; n=4).

**100 μm**
