# Supplementary figures and images for: SNAIL is a key regulator of alveolar rhabdomyosarcoma tumor growth and differentiation through repression of MYF5 and MYOD function
Source: Cell Death Dis. 2018 May 29;9(6):643. doi: 10.1038/s41419-018-0693-8 (PMC5974324; doi:10.1038/s41419-018-0693-8)

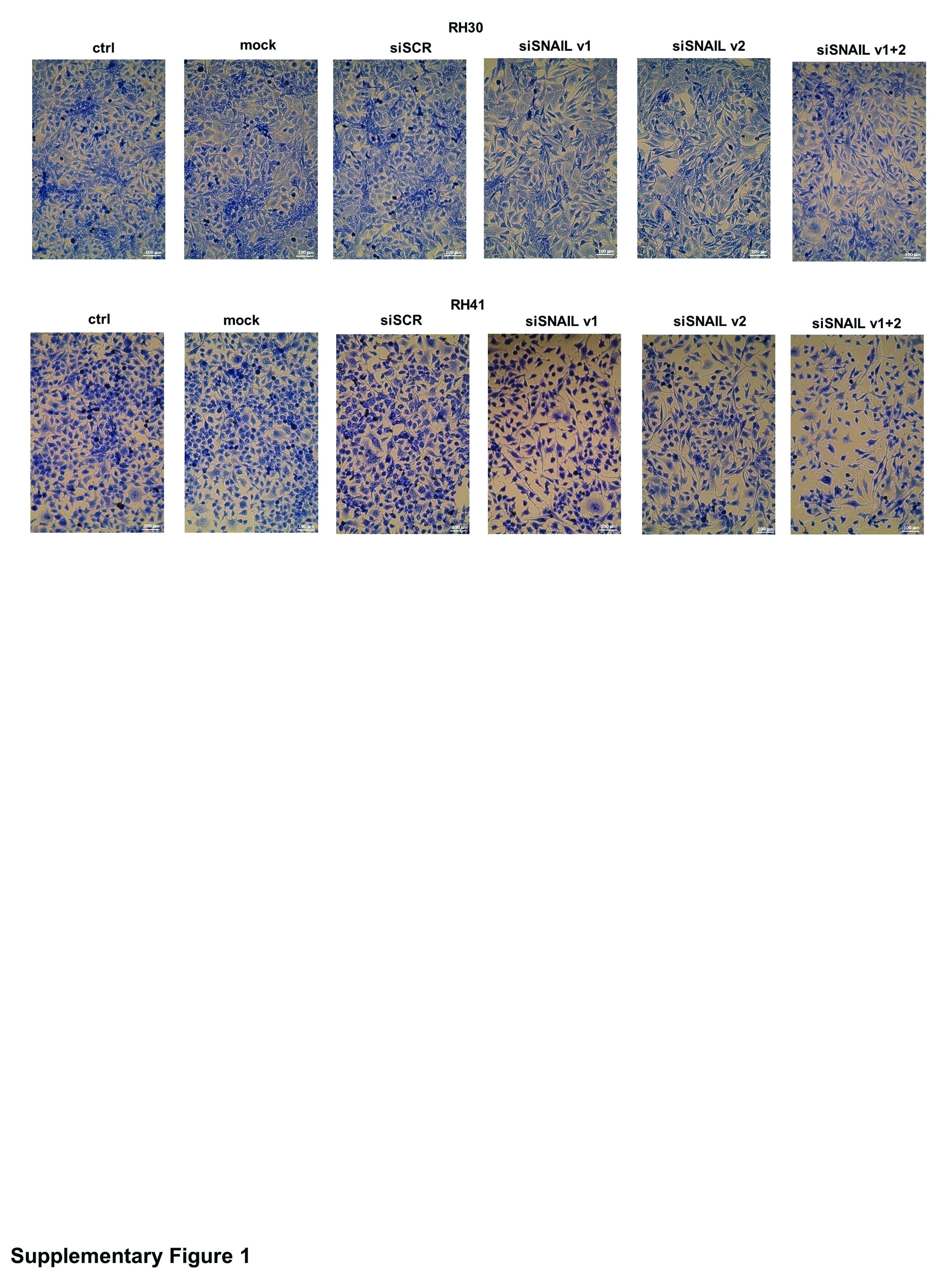

Supplement: Supplementary file 2 — Supplementary Figure 1 [file 41419_2018_693_MOESM2_ESM.tif]

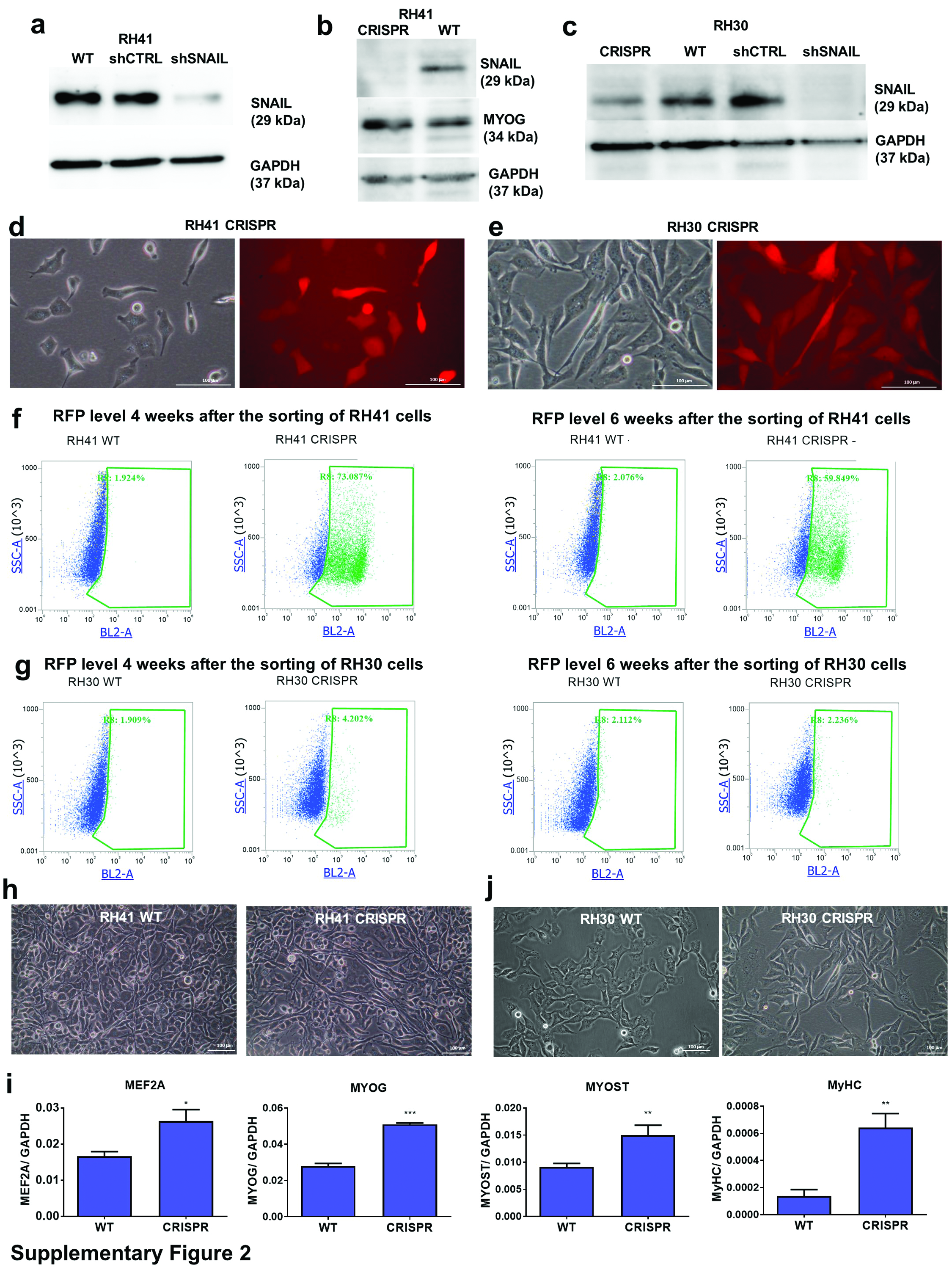

Supplement: Supplementary file 3 — Supplementary Figure 2 [file 41419_2018_693_MOESM3_ESM.tif]
